# Causes and Challenges Regarding Explantation and Reimplantation in Pediatric Cochlear Implant Surgery: A Retrospective Analysis

**DOI:** 10.3390/medicina61091519

**Published:** 2025-08-25

**Authors:** Dan-Cristian Gheorghe, Mihai Dumitru, Adina Zamfir-Chiru-Anton

**Affiliations:** 1 Marie Curie Emergency Hospital for Children, 077120 Bucharest, Romania; dan.gheorghe@umfcd.ro; 2 Department of ENT, Carol Davila University of Medicine and Pharmacy, 050474 Bucharest, Romania; zamfiradina@yahoo.com; 3 ENT Department, Bucharest University Emergency Hospital, 050098 Bucharest, Romania; 4 Grigore Alexandrescu Emergency Hospital for Children, 011743 Bucharest, Romania

**Keywords:** cochlear implants, reimplantation, pediatric

## Abstract

*Background and Objectives:* Cochlear implantation (CI) is a surgical procedure that offers significant benefits to individuals with sensorineural hearing loss, particularly in pediatric patients, as it can prevent long-term cognitive impairment. Despite the devices being designed for lifelong use, complications may necessitate explantation and subsequent reimplantation. *Materials and Methods*: Our retrospective study analyzes the incidence and causes of such procedures in pediatric CI patients over a period of 15 years, from May 2009 to June 2025. The study included patients aged between 8 months and 17 years, recording their age, the manufacturers of their first and second implants, the reasons for explantation and reimplantation, and the type of electrode array used during the second surgery. *Results:* During the study period, a total of 440 cochlear implantations were performed in our department. The primary causes of explantation in our study group were device hardware failures in 2.27% of cases, seromas over the implant body or antenna in 0.68% of cases, spontaneous extrusion in 0.22% of cases, and local trauma with electrode displacement in 0.22% of cases. The study confirmed that hardware failures were the most common reason for reimplantation, with an incidence influenced by the device manufacturer and the extent of trauma to the device. Surgical observations highlight the challenges regarding electrode reimplantation and available electrode choices for the surgeon. *Conclusions*: The use of superior materials and advanced research in manufacturing can enhance implant reliability and reduce the number of surgical procedures required in the long term for pediatric patients. Any type of electrode array can be utilized in reimplantations if meticulous surgical techniques are applied.

## 1. Introduction

Congenital sensorineural hearing loss manifests diverse incidence rates across different regions of the globe, occurring in approximately 1–3 per 1000 newborn children [1,2]. Estimating its prevalence presents a challenge, but the routine implementation of neonatal screening tests facilitates the formulation of certain predictions [3]. Congenital hearing loss can arise from various etiologies, including genetic causes [4], perinatal infections such as cytomegalovirus (CMV) [5], and inner-ear malformations (IEMs) [6]. Delayed management of hearing loss in affected children can have detrimental effects on their speech and emotional development [7]. As the number of children diagnosed with hearing loss and taken in charge increases, a societal burden is anticipated [8]. Furthermore, other children may develop acquired hearing losses throughout their lives, also attributed to genetic causes [9].

Cochlear implantation (CI) is a surgical procedure that offers substantial benefits to individuals with sensorineural hearing loss. The incidence of CI surgery has increased due to earlier diagnosis and a broader range of indications, including unilateral deafness [10]. In pediatric patients, CI can prevent long-term cognitive impairment and ensure their normal social development [11,12,13]. However, optimal outcomes are achieved when the procedure is performed at younger ages, ideally during infancy [14]. The implanted devices are designed to be lifelong and to enable normal hearing behavior in their recipients. Complication rates following CI surgery are relatively low if the operations are performed by skilled otologic surgeons [15]. Nevertheless, due to the anticipated longer lifespan of a child, the risk of implant malfunction or other associated complications may necessitate explantation procedures in certain cases, potentially followed by the subsequent reimplantation of another device [16]. Issues associated with the wear or aging of cochlear implants are currently being discussed, and more than one revision CI surgery can be anticipated, particularly for pediatric patients [17].

In this retrospective study, we present our experience regarding the incidence and causes of such procedures for the patients who underwent surgery in our department. We also emphasize specific surgical aspects pertaining to the types of electrode arrays that can be utilized in these situations and the challenges encountered when performing cochlear reimplantation surgery.

## 2. Materials and Methods

A retrospective study was conducted to analyze the outcomes of pediatric patients who underwent cochlear implantation (CI) surgery at the ENT department of our institution. The study included all patients who underwent CI surgery, covering a period of over 15 years, from March 2009 to June 2025. Devices manufactured by four companies were used in our patient cohort: Cochlear, Medel, Advanced Bionics, and Oticon/Neurelec. Clinical information was reviewed through electronic (InfoWorld, Hospital Manager software) and paper medical records. The type of device failure was determined after audiometric, electrophysiologic, and integrity tests were performed by experienced technicians and audiologists.

Patient selection criteria encompassed individuals under the age of 18, including those with a cochlear implant malfunction as confirmed by the audiology department. Additionally, patients exhibiting clinical symptoms of local complications, such as persistent seroma, skin necrosis, implant extrusion, or CI migration from its designated location, were eligible for inclusion.

The surgical technique employed for initial CI surgery involved a postauricular incision. Following cortical mastoidectomy, a posterior tympanotomy was performed. Electrode array insertion into the cochlea was achieved through cochleostomy (2009–2015) or round window (2015–2025). The CI body was secured by the periosteal lining over the device body, which was placed in a meticulously drilled bed on the external surface of the temporal squamous bone. Electrode array fixation was performed with glass ionomer cement in cases where there was a propensity for the array to protrude from its inserted position within the scala tympani. Mastoid obliteration was not performed at the conclusion of CI surgery; instead, the mastoidectomy was closed using its periosteum. Routine electrode position verification after CI surgery is not customary, as intraoperative measurements of implant parameters are consistently conducted using software provided by each manufacturer. For Cochlear implants, the SmartNav device has been utilized since 2022 for both initial and reimplantation procedures to monitor electrode placement within the inner ear. During implant removal, meticulous drilling and dissection of the electrode arrays from their ossifying course over the mastoid process were undertaken to preserve the mechanical integrity of the devices (a single component), as per the manufacturer’s request.

All 440 CI surgeries were performed by the same surgeon.

We documented each patient’s age, the manufacturer and type of their initial implant and the second implant, the reasons for the first implant’s removal, the dates of initial and subsequent revision surgeries, and the electrode array types utilized during both operations. Surgical records from the operating room were reviewed to verify the types of devices used for reimplantation.

Videos were recorded for documentation purposes, and computed tomography (CT) scanning was conducted postoperatively to confirm the correct positioning of the electrodes within the cochlea, when needed. Visual examinations of the scan images and 3D reconstructions were performed using Falcon MD software (version 5.0.1). Audiograms were obtained from reimplanted patients whenever feasible.

Comprehensive testing of cochlear implant devices was conducted in those instances where the auditory verbal performance of patients was deemed unsuitable and their school performance deteriorated over time, in the absence of any other potential concomitant or explanatory conditions. Parental involvement and school contacts substantially contributed to the identification of potential complications and CI malfunction.

## 3. Results

Between May 2009 and June 2025, a total of 440 cochlear implantations were performed by a single surgeon in our department. Of these, 16 patients underwent explantation, while only 15 underwent reimplantation. The age of patients at the time of reimplantation ranged from 4 to 17 years. The reimplantation rate was 3.18% (considering only the 14 patients who underwent surgery in our department). Genetic tests were not performed for the reimplanted cases.

During the reimplantation procedure, we consistently endeavored to extract CI devices in a single piece. However, this objective was only achieved in two instances. The primary reasons for these limited successes were the complete ossification surrounding the electrode array course and the implant body, as well as the fibrous fixation of the array at the entrance into the cochlea. Consequently, we have not presented electronic reports detailing the removed CIs. Ossification was observed in all patients who underwent surgery for cochlear reimplantation, encompassing both the implant components and the drilled mastoidectomy opening.

The primary causes of explantation in our series were device hardware failures in 10 cases, accounting for 66.67% of all necessary reimplantations performed and 2.27% of all our cochlear-implanted patients (see Table 1).

One of the challenging hardware failure cases also presented with a tympanomastoid cholesteatoma. Consequently, the device was replaced along with the performance of a subtotal petrosectomy. Despite the concurrent medical condition, the patient was still categorized as having a device hardware failure.

Three cases of seromas (infections) were recorded on or near the implant body or antenna (18.75% of our study cases), resulting in an incidence of 0.68% among all our cochlear implant patients. One patient experienced spontaneous cochlear implant extrusion after repeated aspirations and extensive local infection of her seroma. Two others underwent reimplantation due to persistent local conditions despite medical management and the inability of those patients to use their sound processors.

One patient experienced spontaneous extrusion of the implant following primary cochlear implant surgery at another hospital, despite the absence of any subsequent operations or trauma after the initial procedure (6.67% of our total cochlear reimplantation procedures). Two stages of surgical approach were subsequently decided and performed.

One case exhibited spontaneous migration of the device, resulting in both implant body and electrode array displacement, during early postoperative cochlear implant surgery because of local trauma and early extensive epicranial hematoma formation.

One patient, who had the CI implanted abroad, requested device removal due to limited or no usage.

## 4. Discussion

Although cochlear implant surgery has yielded beneficial outcomes in pediatric patients with sensorineural hearing loss, it does carry potential medical and surgical risks. As reported in the published literature, the incidence of complications associated with CI surgery is relatively low [11,18]. Several studies have addressed complications arising from CI surgery and CI revision procedures [19,20,21]. However, there are discrepancies among different published papers regarding the precise and systematic comparison of complications among various surgical centers [15,18,22,23]. Furthermore, the objective delineating criteria for categorizing complications as major or minor are lacking, which hinders the comprehensive review of this topic [22].

The European Consensus Statement on Cochlea Implant Faults and Explants (ECSCIFE) provides a framework for reporting complications that may necessitate cochlear reimplantations (Table 2) [24].

For presenting purposes, most reporting articles on cochlear implant revision surgery discuss device-related causes and medical (surgical) causes as the reasons for CI reimplantation. Notably, certain supplementary medical conditions and social factors can contribute to the number of cochlear implants that require revision or reimplantation surgery (e.g., local trauma, pneumococcal vaccination) [25]. Additionally, it appears that the reliability of the implanted devices significantly impacts the number of reimplantations reported globally [20,26].

It is also important to consider the concept of CI soft failure, which refers to normal device functioning but with abnormal responses from the patient to electrical stimulation of the cochlea. Audiologists have occasionally reported moderate or minor alterations in the functioning parameters of cochlear implant electrodes in association with middle- or inner-ear diseases [27]. While this is a debatable topic, we did not deem it necessary to use reimplantations in such cases. All our revision surgeries addressed CI hardware failures. Audiologic results after revision CI surgery appear uniformly positive, with auditory thresholds that are comparable with or even superior to those achieved with the older devices [17]. Therefore, it is tempting to largely accept more cases for reimplantation as technology advances. However, the medical, surgical, emotional, and financial burdens associated with CI reimplantation surgery still limit its use to patients who are unable to use their implants at all.

Device hardware failure emerged as the primary cause of implant revision CI surgery in our patient cohort, accounting for 66.7% of cases who had undergone surgery. The rate of hardware failure in our revision surgery (2.27%) is comparable with that reported by other centers. Numerous authors have consistently identified device failure as the leading cause of CI revisions, albeit with varying rates among their patient populations, ranging from 1.37 to 3.32% [19,28,29]. The incidence of hardware failures can be influenced by various factors, including the device manufacturer and the extent of postoperative trauma to the cochlear implant [16]. The malfunction of the implants was identified due to the poor auditory–verbal performance of some patients observed by our speech therapists and audiologists during follow-up fitting sessions and confirmed by parental and other educational sources.

In our implant surgery practice, we employ all four major CI manufacturers’ devices, albeit with varying proportions. Notably, from the devices that exhibited hardware failure, Neurelec/Oticon manufactured seven, Medel manufactured five, Cochlear manufactured two, and Advanced Bionics (AB) manufactured two. Other authors have also reported differences in CI hardware failure rates [20,28]. In our series, one implant (AB) was removed due to the patient’s request for low or non-use, despite the device functioning as intended.

Based on our preoperative audiologic findings, in eight cases (80%) of CI hardware failure, half or more of the total number of electrodes ceased functioning. In two cases (20%), no CI activity was elicited. We were unable to establish a correlation between the number of remaining functioning electrodes and the device’s age. A recent article raised concerns about this topic, which has significant implications for pediatric practice [17].

Medical and surgical complications contribute to the increasing number of cases requiring reimplantation of cochlear implants. The incidence of local flap infections and device extrusion varies across studies, ranging from 1.9 to 5.9% [21,26,28,30], and typically necessitates reimplantation. In our cohort, the incidence of surgical or medical complications that led to the revision of the CI was lower (0.68%). We hypothesize that experienced single-surgeon implantation techniques may contribute to optimal and consistent outcomes. However, some confusion elements can complicate the interpretation of data, since many CI centers (like ours) perform such procedures on patients who have initially received cochlear implants elsewhere [19]. Therefore, reported data are merged and cannot be directly correlated with the first CI surgery or the operative technique. Instead, publications that report the experience of a single center often present similar rates of medical and surgical complications, as observed in our series [20].

Device infection is reported in approximately 1.62 to 2.3% cases [29,31]. In our patient cohort, the incidence rate was lower than that reported in other published data (0.68%). Additionally, we did not record any spontaneous extrusion. In one case, it occurred after repeated local punctures followed by a local suppurative process. The only true implant extrusion case observed in our series was a child implanted at another CI center. Therefore, we can consider our patient as iatrogenic. This implies a 0.22% rate of device extrusion in our CI patient cohort.

Managing complications associated with skin flaps following CI surgery can be particularly challenging, especially when these complications arise significantly later after the initial surgical procedure. Various methods have been reported to prevent reimplantation in such cases [32,33]. In our experience, local and/or general management with antibiotics and corticosteroids can be effective if complications are diagnosed early enough after surgery. However, as the duration from first implantation grows, it becomes increasingly difficult to provide efficient solutions and to avoid the need for another surgery. Two cases responded favorably to this approach, and we were able to prevent reimplantation. These cases are not included in this study.

**Figure 1:**
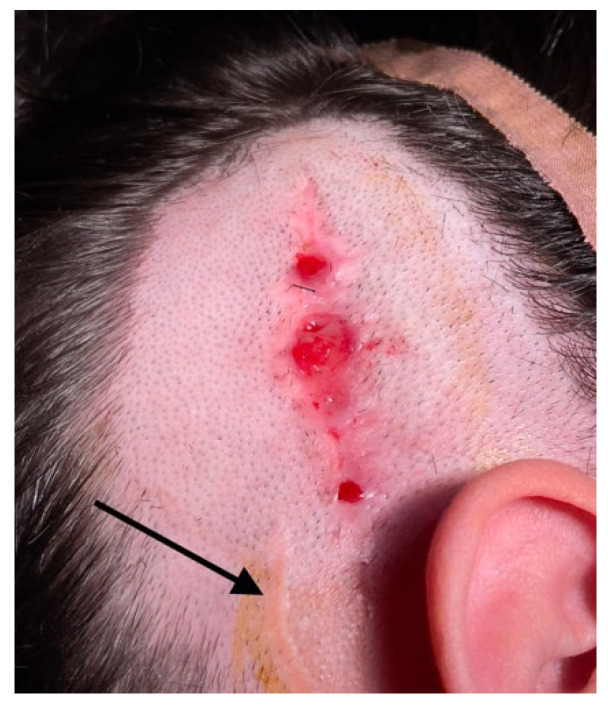
Skin breakdown occurred over the previous implant site, but in a different location from the surgical incision (black arrow). The device had already been removed two months prior, necessitating skin repair through excision and suture three times before complete healing could be achieved.

In cases where skin flap complications affect the implant body and antenna, reimplantation can be challenging due to the difficulty in achieving a stable covering over the implanted device. Skin breaks and granulations can hinder revision surgery (Figure 1). While the specific causes of skin healing abnormalities in one of our cases remain unknown, the recurrence of lesions (requiring three skin excisions and repairs) suggests a potential allergic reaction to the CI covering material. This hypothesis has been proposed by other authors [34]. In our patient, removal by drilling a fine layer of bone over the temporal bone resulted in long-term healthy skin. For similar cases, two-stage surgery can always be considered to prevent postoperative complications and enhance the surgical success rate. To date, no local flap complications have been observed in our operated extrusion cases following reimplantation.

During reimplantation, we identified additional potential electrode choice options and surgical challenges. In cases of cochlear implants equipped with ring-sealing electrodes (from Oticon/Neurelec), electrode extraction proved challenging. The solution involved meticulously removing the fibrous tissues surrounding the CI electrode array at the cochlear entrance (above the electrode rings). We did not record any remnants of the previous electrode array within the scala tympani after any of our explantations. Precise and delicate electrode removal ensured its integrity and patient recovery. After removing the old electrode, a clear view of the scala tympani (a large, clearly open, fibrous canal) should always be discernible. Disruption of the fibrous tissues at the cochlear opening, as observed in other cases [35], could render the reinsertion impossible. In one instance, we successfully resolved this issue by removing a small rim of the fibrous sheath located within the scala tympani, using a small hook inserted through the round window.

**Figure 2:**
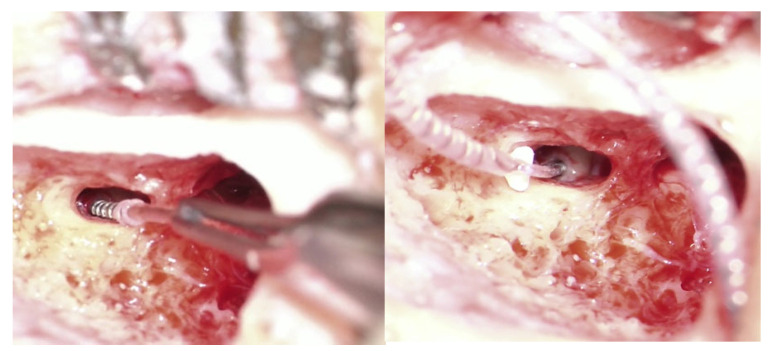
Reimplantation with a modiolar electrode array (Cochlear CI632—**right image**) following the removal of a lateral wall type from a different device manufacturer (Neurelec, Digisonic SP—**left image**).

The optimal electrode array selection for reimplantation has been a subject of ongoing debate [36,37]. Lateral wall electrodes have been successfully utilized after modiolar-type extraction, even employing longer dimensions than the original ones [36]. From an anatomical perspective, highly flexible and soft electrode arrays appear to be the most suitable for this purpose [37]. Consequently, lateral wall and extremely thin electrode arrays are favored by various authors. Our experience demonstrates that any electrode array can be inserted into the scala tympani, provided that its opening remains undisturbed following the removal of the previous implant electrode. In three instances, we employed a different electrode array type compared with the original one. Two patients received slim modiolar (Cochlear CI632) CIs after removal of a Neurelec-manufactured implant (Figure 2) and after the explantation of a slim straight Nucleus from Cochlear (CI622). Complete insertion was achieved in all reimplanted cases, although some authors report lower success rates [30].

**Figure 3:**
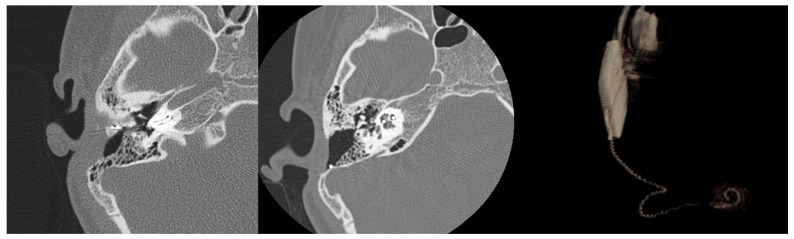
CT scan images depicting the positioning of the old electrode array CI622 (**left image**) and the new one after reimplantation with a slim modiolar CI632 (**middle** and **right images**). The reconstruction (via volume rendering) of the electrode within the cochlea facilitates visualization of the proper positioning of the new electrode in the scala tympani.

**Figure 4:**
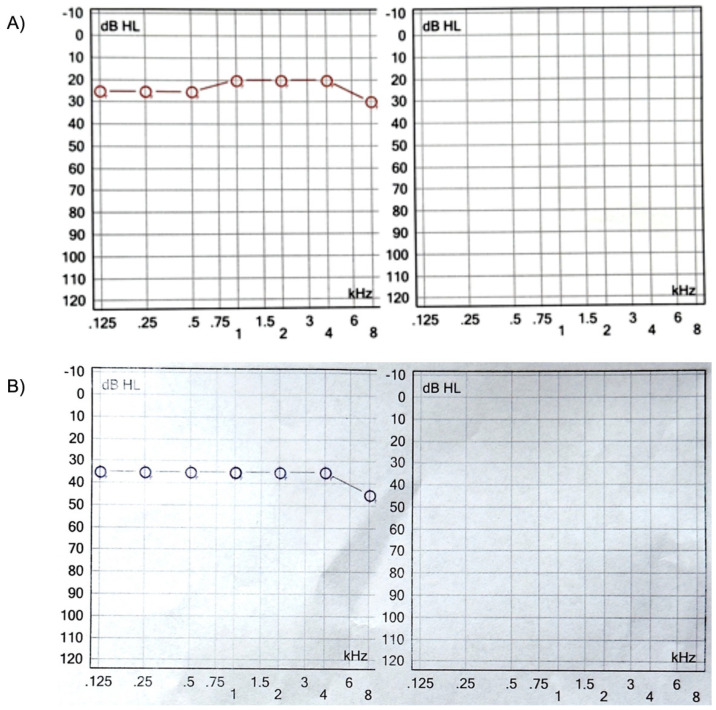
(**A**). Audiogram of the CI622 reimplanted patient with a different electrode array (CI632) four months after surgery. (**B**). Audiogram of the patient implanted with the CI632 after removing a Medel Flex 24, one month after activation.

Four months after reimplantation, the previous Nucleus slim straight (CI622) patient underwent computed tomography (CT) imaging and audiologic examinations, which demonstrated the correct placement of the device within the cochlea and a favorable hearing outcome (Figure 3 and Figure 4).

**Figure 5:**
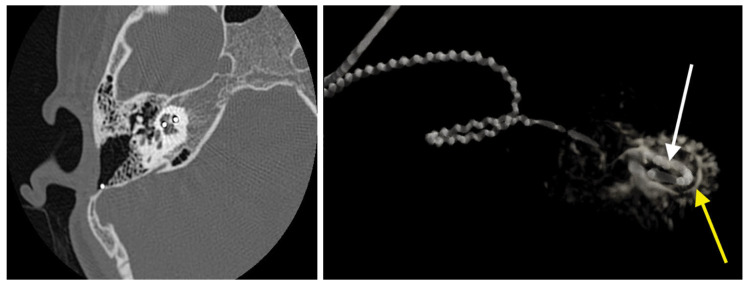
CT scan images of a case illustrating the positioning of the CI632. The reimplanted electrode array demonstrates a more lateral orientation relative to the modiolus (white arrow), although it does not make contact with the lateral wall of the scala tympani (yellow arrow).

It is challenging to provide an exact assessment of the newly inserted electrode array’s position within the scala tympani. Image analysis and the 3D volume rendering of the CT scan images indicate an intermediate position of the CI632 array in the scala tympani (lateral wall versus perimodiolar). This suggests a certain degree of elasticity or flexibility of the fibrous sheath within the inner ear after explantation, potentially explaining the possible use of various electrode arrays for reimplantation surgery (Figure 5).

**Figure 6:**
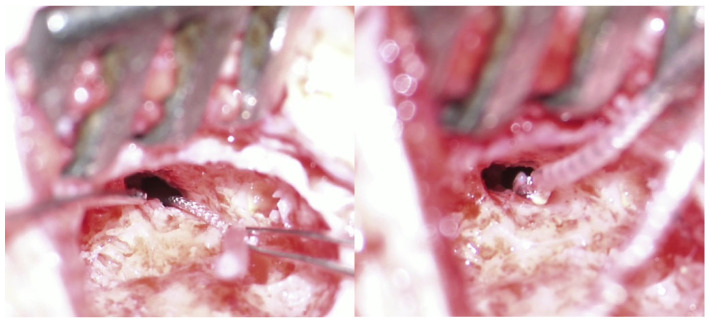
Removal of a lateral wall electrode array (Medel Flex 24—**left image**) and reimplantation with a slim modiolar (Cochlear CI632—**right image**) device.

Following the explantation of a Medel Flex 24 electrode, a slim modiolar CI632 was inserted (Figure 6).

While some may express reservations regarding the use of modiolar-hugging electrodes due to their reduced flexibility and potential for causing damage to the delicate structures of the modiolus in the event of multiple insertions, it is important to acknowledge the significant advantages that they offer. These include enhanced stimulation of the cochlear neurons, ease of insertion, and improved long-term stability, which can undoubtedly justify their continued use [37].

Despite the development of effective surgical techniques over time, displaced CI electrodes have been reported [38,39]. Some papers state this potential complication may even occur a long time after the initial CI surgery and can be attributed to head trauma [40]. We documented a case where the cochlear implant (CI) migrated early postoperatively due to blunt trauma to the surgical site, resulting in its displacement and complete extrusion of the electrode array from the cochlea during the immediate postoperative period. This occurrence was linked to the lack of parental supervision. A large epicranial hematoma was noted clinically and the plain X-ray demonstrated device migration and complete extrusion of the electrode array from the cochlea. Early wound reopening facilitated reinsertion of the electrode within the cochlea and subsequent fixation of the device. This case emphasizes the significance of two surgical objectives during any CI surgery: meticulously drilling an implant bed to ensure device stability and achieving thorough hemostasis of the surgical site. The mastoid cavity and surgical field should be thoroughly verified for potential bleeding and cerebrospinal fluid (CSF) leakage to prevent postoperative hematoma or meningitis. Additionally, some patients may require postoperative sedation based on the available postsurgical supervision to prevent head trauma. In our opinion, the risk of electrode displacement after long periods of time following CI surgery cannot be attributed solely to trauma, as ossification processes undoubtedly fixate the electrodes and body implant to the temporal bone. It is more likely that head growth in pediatric patients could contribute to the risk of partial extrusion of the electrode array from the scala tympani and deterioration of the CI benefit upon long-term follow-up. Regular audiologic monitoring can be beneficial and raise concerns in selected cases [41].

During revision CI surgery, preparing an adequate implant bed can be challenging or time-consuming due to pre-existing anatomic limitations. The pre-existing ossification over the old CI body and a previously drilled implant well that has thinned the cranial bone can hinder new implant stabilization. Additionally, the need to remove the fibrous sheath coating of the old device complicates the creation of a tight periosteal pocket (which could contribute to device fixation) and increases the difficulty regarding new CI surgical stabilization. In rare cases, periosteal sutures or glass ionomer cement (Ketac^®^ Cem) have been utilized to secure devices and the electrode arrays, ensuring implant fixation in some patients.

As previously discussed, in our facility, one case necessitated the removal of the cochlear implant due to limited or no usage. The patient, a 13-year-old girl who had undergone her first CI surgery abroad, was utilizing sign language within her community. Despite our attempts to persuade her to refrain from surgical explantation, we ultimately decided to explant the device completely. Consequently, we believe it prudent to avoid initial CI surgery in such cases, as it does not contribute to societal benefits and complicates the allocation of medical resources.

The prevention of explantations in cases with normal cochlear implant functionality can be accomplished through diverse approaches. The early application of local antibiotic ointments can facilitate the closure of minor skin defects during the delayed healing process. Puncture with suction of a seroma over the implant antenna can result in complete resolution, although it carries the potential risk of infection and subsequent CI extrusion.

In our study, we excluded patients with inner ear malformations (IEMs) because none of those cases required device replacement. We intend to document our experience in the future with instances of reimplantations for IEM patients.

Several limitations are associated with our study. It is retrospective, which may have resulted in the loss of follow-up for some of the explanted and reimplanted patients. We did not present all the audiologic results of our reimplanted patients. As the largest referral center in our country, it is possible that some of our operated cases attended cochlear implant centers closer to their location. Additionally, the use of various manufacturer models of cochlear implants in different proportions in our department for our CI patients complicates the comparison of explantation rates and CI reliability rates between different brands. This topic could be of interest for future studies since the scientific research regarding materials and the techniques promoted by each manufacturer have changed and evolved towards better surgical results. What we regard as most important from our experience is suggesting the possible use of different electrode arrays other than the original one in CI revision surgery. Perimodiolar electrodes are as useful as the lateral type. We did not manage to determine the significance of the fibrous sheath around the explanted cochlear implant. Its histologic examination is performed for each patient, but since the number of cases is still low, we cannot draw clear conclusions yet. We currently lack data demonstrating the cognitive function of our patients. The oldest patient in our series is 18 years old (since the commencement of the CI program in our pediatric ENT department in 2009), and they are preparing for university admission. Published data do not explicitly mention the potential cognitive effects of cochlear implants on children but support earlier intervention [42,43]. Moreover, in our cohort, there is no impact regarding the nationality of the implant recipient, as all implant patients need to be Romanian citizens to be part of the National Cochlear Implant Program. This gives our data an increased level of consistency compared with other situations where the patient’s ethnic background impacted on the percentage of hearing hours and cochlear implant device use [44]. Numerous pathways, including ischemia–reperfusion injury, inflammation, hair cell death, and mitochondrial dysfunction, are involved in the mechanisms behind oxidative stress-induced hearing loss [45]. Our group study could be further included in systematic reviews focusing on pediatric cases.

## 5. Conclusions

Cochlear implant hardware failure is the primary reason for reimplantation surgery in our department. Our incidence rate is comparable with that reported in other studies. The disparate rates of CI reimplantation underscore the necessity of manufacturers employing superior materials in their manufacturing processes and the incorporation of contemporary research to augment implant reliability, thereby mitigating the number of surgical procedures necessitated for pediatric patients. We observed fewer device and medical failure incidence complications leading to reimplantations than those in the actual literature data. It is challenging to determine whether this represents an effect of the surgical technique or the experience of our CI surgeon. As demonstrated, any type of electrode array can be utilized in reimplantation surgery provided meticulous techniques are employed. Complete insertion can be consistently achieved if the total length of the cochlea is respected. Psychological or psychiatric evaluation of patients should always be an integral part of the first CI surgical preoperative evaluation to prevent non-use cases and redundant explantations.

## Figures and Tables

**Table 1:** Causes of cochlear implant revision.

Cause of Explantation/ Reimplantation	No. of Implants	Percentage of CI Patients	Observations
Hardware failure	10	2.27%	All brands of CI manufacturer
Implant seroma/infection	3	0.68%	1 more case was solved with AB and corticoids (not reported here)
CI migration	1	0.22%	Poor parental care after surgery
CI extrusion	1	0.22%	First CI surgery elsewhere
Little/no use of CI	1	0.22%	Only explanted

**Table 2:** European Consensus Statement on Cochlea Implant Faults and Explants.

Classification	Name	Number of Cases (Our Series)
A	Device working correctly	2
B1	Alterations in device characteristics	8
B2	Alterations in device performance	-
C	Device faults	2
D	Medical causes	4
E	Interrupted follow-up	-

## Data Availability

The data are not publicly available due to privacy or ethical restrictions.

## Abbreviations

The following abbreviations are used in this manuscript:

CI	Cochlear implantation
CT	Computed tomography
CSF	Cerebro-spinal fluid
IEM	Inner-ear malformations

## References

[1] Feder K.P., Michaud D., McNamee J., Fitzpatrick E., Ramage-Morin P., Beauregard Y. (2017). Prevalence of Hearing Loss Among a Representative Sample of Canadian Children and Adolescents, 3 to 19 Years of Age. Ear Hear..

[2] Smith R.J.H., Bale J.F., White K.R. (2005). Sensorineural hearing loss in children. Lancet.

[3] Yoshinaga-Itano C. (2003). From Screening to Early Identification and Intervention: Discovering Predictors to Successful Outcomes for Children With Significant Hearing Loss. J. Deaf. Stud. Deaf. Educ..

[4] van Beeck Calkoen E.A., Engel M.S.D., van de Kamp J.M., Yntema H.G., Goverts S.T., Mulder M.F., Merkus P., Hensen E.F. (2019). The etiological evaluation of sensorineural hearing loss in children. Eur. J. Pediatr..

[5] Fowler K.B., Boppana S.B. (2006). Congenital cytomegalovirus (CMV) infection and hearing deficit. J. Clin. Virol..

[6] Sennaroglu L., Bajin M.D. (2017). Classification and Current Management of Inner Ear Malformations. Balk. Med. J..

[7] Tomblin J.B., Harrison M., Ambrose S.E., Walker E.A., Oleson J.J., Moeller M.P. (2015). Language Outcomes in Young Children with Mild to Severe Hearing Loss. Ear Hear..

[8] Dikeç G., Türk E., Yüksel E., Çelebi K., Özdemir M. (2023). Experiences of Hearing Parents of Children with Hearing Loss: A Qualitative Study. Children.

[9] Marazita M.L., Ploughman L.M., Rawlings B., Remington E., Arnos K.S., Nance W.E. (1993). Genetic epidemiological studies of early-onset deafness in the U.S. school-age population. Am. J. Med. Genet..

[10] Lassig A.-A.D., Zwolan T.A., Telian S.A. (2005). Cochlear Implant Failures and Revision. Otol. Neurotol..

[11] Hoff S., Ryan M., Thomas D., Tournis E., Kenny H., Hajduk J., Young N.M. (2019). Safety and Effectiveness of Cochlear Implantation of Young Children, Including Those With Complicating Conditions. Otol. Neurotol..

[12] Sharma S.D., Cushing S.L., Papsin B.C., Gordon K.A. (2020). Hearing and speech benefits of cochlear implantation in children: A review of the literature. Int. J. Pediatr. Otorhinolaryngol..

[13] Tarkan O., Tuncer U., Ozdemir S., Surmelioglu O., Cetik F., Kiroglu M., Kayikcioglu E., Kara K. (2013). Surgical and medical management for complications in 475 consecutive pediatric cochlear implantations. Int. J. Pediatr. Otorhinolaryngol..

[14] Miyamoto R.T., Houston D.M., Bergeson T. (2005). Cochlear implantation in deaf infants. Laryngoscope.

[15] Daneshi A., Ajalloueyan M., Ghasemi M.M., Hashemi B.S., Emamjome H., Farhadi M., Ajalloueyan Z. (2015). Complications in a series of 4400 paediatric cochlear implantation. Int. J. Pediatr. Otorhinolaryngol..

[16] Barrera S., Kerby E., Gonzalez V., Carron J. (2024). Surgical and audiologic outcomes following revision cochlear implantation in children. Am. J. Otolaryngol..

[17] Soloperto D., Confuorto G., Dallari V., Sacchetto L., Carner M., Monzani D., Nocini R. (2025). Long-Term Outcomes Following Cochlear Implantation: Device “Aging” and Hearing Performance. Audiol. Res..

[18] Parent V., Codet M., Aubry K., Bordure P., Bozorg-Grayeli A., Deguine O., Eyermann C., Franco-Vidal V., Guevara N., Karkas A. (2020). The French Cochlear Implant Registry (EPIIC): Cochlear implantation complications. Eur. Ann. Otorhinolaryngol. Head Neck Dis..

[19] Chen Z., Bi Q., Lv Y., Li Y., Yang W., Xu X., Li Y. (2024). Cochlear reimplantation outcomes over 20 years: Expertise in reimplantation surgery and auditory-speech rehabilitation. Am. J. Otolaryngol..

[20] Lane C., Zimmerman K., Agrawal S., Parnes L. (2020). Cochlear implant failures and reimplantation: A 30-year analysis and literature review. Laryngoscope.

[21] Yeung J., Griffin A., Newton S., Kenna M., Licameli G.R. (2018). Revision cochlear implant surgery in children: Surgical and audiological outcomes. Laryngoscope.

[22] Googe B.J., Carron J.D. (2016). Analyzing complications of minimally invasive pediatric cochlear implantation: A review of 248 implantations. Am. J. Otolaryngol..

[23] Sivam S.K., Syms C.A., King S.M., Perry B.P. (2017). Consideration for routine outpatient pediatric cochlear implantation: A retrospective chart review of immediate post-operative complications. Int. J. Pediatr. Otorhinolaryngol..

[24] (2005). European consensus statement on cochlear implant failures and explantations. Otol. Neurotol..

[25] Eskander A., Gordon K.A., Kadhim L., Papaioannou V., Cushing S.L., James A.L., Papsin B.C. (2011). Low Pediatric Cochlear Implant Failure Rate: Contributing Factors in Large-Volume Practice. Arch. Otolaryngol.–Head Neck Surg..

[26] Marlowe A.L., Chinnici J.E., Rivas A., Niparko J.K., Francis H.W. (2010). Revision Cochlear Implant Surgery in Children: The Johns Hopkins Experience. Otol. Neurotol..

[27] Sainz M., Roldan C., de la Torre A., Gonzalez M.V., Ruiz J.M. (2003). Transitory alterations of the electrode impedances in cochlear implants associated to middle and inner ear diseases. Int. Congr. Ser..

[28] Hou V., Tellez P., Fandino M., Ospina J., Chia R., Bergstrom R., Riding K., Kozak J., Kozak E., Pauwels J. (2023). Pediatric cochlear implant explantation and reimplantation over a 32-year period. Int. J. Pediatr. Otorhinolaryngol..

[29] Trozzi M., Powell H.R., Toma S., Ahmed W., Jephson C.G., Rajput K., Cochrane L.A. (2015). Cochlear re-implant rates in children: 20 years experience in a quaternary paediatric cochlear implant centre. Eur. Arch. Otorhinolaryngol..

[30] Sterkers F., Merklen F., Piron J.P., Vieu A., Venail F., Uziel A., Mondain M. (2015). Outcomes after cochlear reimplantation in children. Int. J. Pediatr. Otorhinolaryngol..

[31] Low W.K., Rangabashyam M., Wang F. (2014). Management of major post-cochlear implant wound infections. Eur. Arch. Otorhinolaryngol..

[32] Geraghty M., Fagan P., Moisidis E. (2014). Management of cochlear implant device extrusion: Case series and literature review. J. Laryngol. Otol..

[33] Karimnejad K., Akhter A.S., Walen S.G., Mikulec A.A. (2017). The temporoparietal fascia flap for coverage of cochlear reimplantation following extrusion. Int. J. Pediatr. Otorhinolaryngol..

[34] Gutiérrez-Salazar A., Cop C., Osorio-Acosta Á., Borkoski-Barreiro S., Falcón-González J.C., Ramos-Macías Á. (2015). Experience in Cochlear Reimplantation. Descriptive Study of a 20-Year Period. Acta Otorrinolaringol. (Engl. Ed.).

[35] Cosetti M., Roland J.T. (2010). Cochlear implant electrode insertion. Oper. Tech. Otolaryngol. Head Neck Surg..

[36] Lyutenski S., Zellhuber N., Helbig R., James P., Bloching M. (2021). Cochlear reimplantation from mid-scala to lateral wall electrode array: Surgical and hearing outcome. Clin. Case Rep..

[37] Mistrík P., Jolly C., Sieber D., Hochmair I. (2017). Challenging aspects of contemporary cochlear implant electrode array design. World J. Otorhinolaryngol. Head Neck Surg..

[38] Rader T., Baumann U., Stöver T., Weissgerber T., Adel Y., Leinung M., Helbig S. (2016). Management of Cochlear Implant Electrode Migration. Otol. Neurotol..

[39] von Mitzlaff C., Dalbert A., Winklhofer S., Veraguth D., Huber A., Röösli C. (2021). Electrode migration after cochlear implantation. Cochlear Implant. Int..

[40] Benyo S., Saadi R.A., Dornhoffer J.L. (2022). Head trauma and Cochlear implant displacement—A systematic review. Am. J. Otolaryngol..

[41] Chakravorti S., Noble J.H., Gifford R.H., Dawant B.M., O’Connell B.P., Wang J., Labadie R.F. (2019). Further Evidence of the Relationship Between Cochlear Implant Electrode Positioning and Hearing Outcomes. Otol. Neurotol..

[42] Almomani F., Al-Momani M.O., Garadat S., Alqudah S., Kassab M., Hamadneh S., Rauterkus G., Gans R. (2021). Cognitive functioning in Deaf children using Cochlear implants. BMC Pediatr..

[43] Hilviu D., Parola A., Vivaldo S., Di Lisi D., Consolino P., Bosco F. (2021). Children with hearing impairment and early cochlear implant: A pragmatic assessment. Heliyon.

[44] Gagnon E.B., Thompson E.M., Park L.R. (2024). Factors Influencing Pediatric Cochlear Implant Use. Am. J. Audiol..

[45] Maniaci A., La Via L., Lechien J.R., Sangiorgio G., Iannella G., Magliulo G., Pace A., Mat Q., Lavalle S., Lentini M. (2024). Hearing Loss and Oxidative Stress: A Comprehensive Review. Antioxidants.

